# Genomic, Transcriptomic, and Phenotypic Analyses of *Neisseria meningitidis* Isolates from Disease Patients and Their Household Contacts

**DOI:** 10.1128/mSystems.00127-17

**Published:** 2017-11-14

**Authors:** Xiaoyun Ren, David A. Eccles, Gabrielle A. Greig, Jane Clapham, Nicole E. Wheeler, Stinus Lindgreen, Paul P. Gardner, Joanna K. MacKichan

**Affiliations:** a Invasive Pathogens Laboratory, Institute of Environmental Science and Research, Porirua, New Zealand; b Malaghan Institute of Medical Research, Wellington, New Zealand; c School of Biological Sciences, Victoria University of Wellington, Wellington, New Zealand; d School of Biological Sciences, University of Canterbury, Christchurch, New Zealand; e Wellcome Trust Sanger Institute, Hinxton, United Kingdom; f H. Lundbeck A/S, Valby, Denmark; g Centre for Biodiscovery, Victoria University of Wellington, Wellington, New Zealand; G. W. Hooper Research Foundation

**Keywords:** *Neisseria meningitidis*, type IV pili, carriage, household contact

## Abstract

*Neisseria meningitidis* (meningococcus) can cause meningococcal disease, a rapidly progressing and often fatal disease that can occur in previously healthy children. Meningococci are found in healthy carriers, where they reside in the nasopharynx as commensals. While carriage is relatively common, invasive disease, associated with hypervirulent strains, is a comparatively rare event. The basis of increased virulence in some strains is not well understood. New Zealand suffered a protracted meningococcal disease epidemic, from 1991 to 2008. During this time, a household carriage study was carried out in Auckland: household contacts of index meningococcal disease patients were swabbed for isolation of carriage strains. In many households, healthy carriers harbored strains identical, as determined by laboratory typing, to the ones infecting the associated patient. We carried out more-detailed analyses of carriage and disease isolates from a select number of households. We found that isolates, although indistinguishable by laboratory typing methods and likely closely related, had many differences. We identified multiple genome variants and transcriptional differences between isolates. These studies enabled the identification of two new phase-variable genes. We also found that several carriage strains had lost their type IV pili and that this loss correlated with reduced tumor necrosis factor alpha (TNF-α) expression when cultured with epithelial cells. While nonpiliated meningococcal isolates have been previously found in carriage strains, this is the first evidence of an association between type IV pili from meningococci and a proinflammatory epithelial response. We also identified potentially important metabolic differences between carriage and disease isolates, including the sulfate assimilation pathway.

**IMPORTANCE** *Neisseria meningitidis* causes meningococcal disease but is frequently carried in the throats of healthy individuals; the factors that determine whether invasive disease develops are not completely understood. We carried out detailed studies of isolates, collected from patients and their household contacts, to identify differences between commensal throat isolates and those that caused invasive disease. Though isolates were identical by laboratory typing methods, we uncovered many differences in their genomes, in gene expression, and in their interactions with host cells. In particular, we found that several carriage isolates had lost their type IV pili, a surprising finding since pili are often described as essential for colonization. However, loss of type IV pili correlated with reduced secretion of a proinflammatory cytokine, TNF-α, when meningococci were cocultured with human bronchial epithelial cells; hence, the loss of pili could provide an advantage to meningococci, by resulting in a dampened localized host immune response.

## Abstract (precis)

*Neisseria meningitidis* causes meningococcal disease but is frequently carried in the throats of healthy individuals; the factors that determine whether invasive disease develops are not completely understood. We carried out detailed studies of isolates, collected from patients and their household contacts, to identify differences between commensal throat isolates and those that caused invasive disease. Though isolates were identical by laboratory typing methods, we uncovered many differences in their genomes, in gene expression, and in their interactions with host cells. In particular, we found that several carriage isolates had lost their type IV pili, a surprising finding since pili are often described as essential for colonization. However, loss of type IV pili correlated with reduced secretion of a proinflammatory cytokine, TNF-α, when meningococci were cocultured with human bronchial epithelial cells; hence, the loss of pili could provide an advantage to meningococci, by resulting in a dampened localized host immune response.

## INTRODUCTION

*Neisseria meningitidis* is a Gram-negative bacterium and the causative agent of meningococcal disease, which can result in inflammation of the meninges as well as septicemia. This disease is often fatal, even with prompt antibiotic treatment, and often causes long-term sequelae (1). Meningococcal disease can occur sporadically or in epidemics that affect millions of people, especially children, worldwide. Despite the seriousness of the invasive disease, *N. meningitidis* comprises part of the normal nasopharyngeal flora of healthy individuals; host invasion, resulting in septicemia and meningitis, is a relatively rare event. Epidemiology reveals that only a small group of hypervirulent lineages cause most meningococcal disease cases in the world (2, 3). From 1991 to 2008, New Zealand experienced a protracted epidemic, with the majority (>85%) of disease cases caused by a single strain type, defined as B:4:P1.7-2,4, belonging to one of two sequence types (STs), ST-42 and ST-154, of the ST-41/44 clonal complex (lineage 3) (4–6). The 2005 introduction of MenNZB, a strain-specific vaccine that elicited protective antibodies to the PorA allele, lowered the number of cases dramatically, until it was discontinued in 2008 (7–10). However, this strain type remains in circulation in New Zealand, having caused >16% (11/68) of total meningococcal cases in 2013 (11). Carriage studies conducted during the epidemic suggested that the New Zealand epidemic strain type is highly virulent but with low transmissibility (12). The genetic basis of this high virulence has not been identified.

Virulence in *N. meningitidis* is thought to be polygenic, with many different genes contributing to invasive disease (13). Comparing the genomes of large numbers of isolates has not given a clear picture of virulence-related genes, due to the extensive genetic diversity among invasive and carriage strains (14). Previous gene expression studies showed large differences in the transcription repertoire between *N. meningitidis* and the related commensal species *Neisseria lactamica* (15), as well as between a disease-associated serogroup B strain (MC58) and a carriage-associated strain (alpha710) upon adhesion to human nasopharyngeal cells (13).

Household crowding has been shown to be a major risk factor for meningococcal disease, and household contact with a meningococcal disease patient is associated with increased risk for invasive disease (16–18). During the New Zealand epidemic, a study of carriage rates among household contacts of patients was carried out in Auckland (12). *N. meningitidis* was cultured from throat swabs from healthy household contacts of an index meningococcal disease patient, within 24 h of disease notification and prior to administration of chemoprophylaxis to contacts. *N. meningitidis* isolates were collected from patient blood or cerebrospinal fluid (CSF). Of 108 *N. meningitidis* carriers, 50% were found to carry the same strain type, as identified by multilocus sequence typing (MLST), serotyping, and *porA* subtyping, as the index patient from their household (12).

Though host and environmental factors play a role in disease development, we asked whether bacterial differences could have also played a role in disease development. We selected several groups of *N. meningitidis* isolates, derived from single households and in most cases indistinguishable by laboratory typing methods, to identify genetic differences potentially important for disease development. Isolates from one household were investigated in greater detail and were found to differ in their interactions with tissue culture host cells. Whole-genome sequencing and transcriptome analysis methods were used to identify variations. We then investigated potential associations between these variants and phenotypes.

## RESULTS

### Whole-genome sequencing of household isolates reveals that they are closely related.

**FIG 1:**
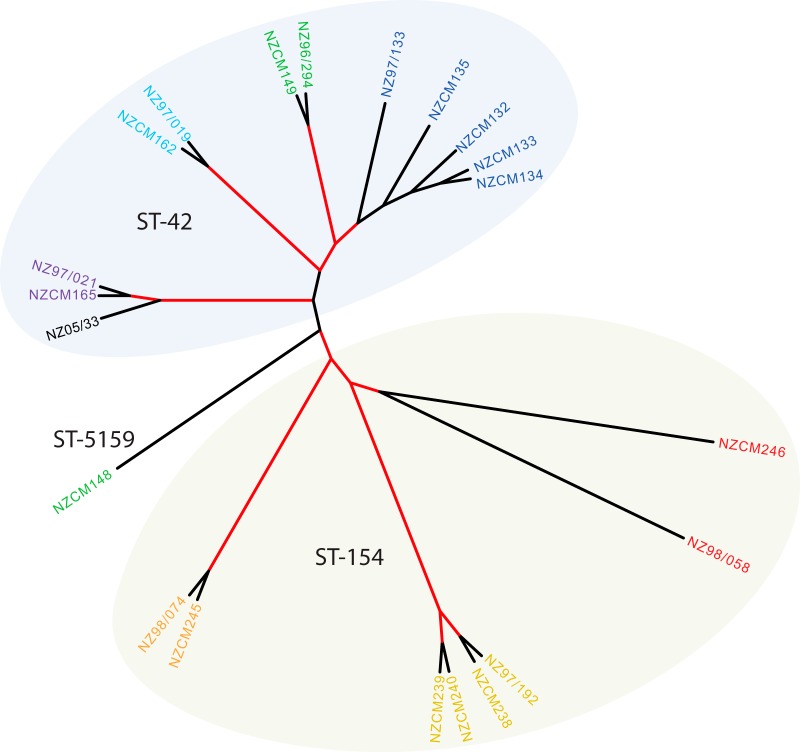
Maximum-likelihood phylogeny of the household isolates from core SNPs. A phylogeny was constructed based on core SNP differences between each isolate and the reference genome of *N. meningitidis* NZ-05/33 (NC_017518.1). A proportional cladogram is presented so that short branches are visible. Branches with greater than 90% (200× bootstrap) support are highlighted in red. Isolates from the same household are shown in the same color. Two ovals indicate the sequence types of the isolates within the two major branches. Isolate NZCM148 from household NZ96/294 belongs to a different clonal complex.

During the height of the New Zealand serogroup B meningococcal epidemic, a study was carried out to assess the prevalence of carriage among household contacts of patients with meningococcal disease (12). A total of 954 contacts of 160 patients agreed to nasopharyngeal swabs; 196 of these contacts (20.5%) were found to be carriers. Isolates from 51 households, including those from healthy subjects and from patients, were subjected to routine MLST and serotyping, revealing that 29 of the 51 households had at least one healthy individual carrying a meningococcal isolate indistinguishable from the invasive isolate from the patient. MLST, based on the nucleotide sequence of 7 housekeeping genes, does not give a full picture of the genetic diversity of the isolates. To address this, we selected 21 isolates, from 7 households, to analyze further with shotgun whole-genome sequencing using Illumina MiSeq technology. The index meningococcal isolates for all 7 households were of the New Zealand epidemic type, with three belonging to ST-154 and four belonging to ST-42 (Table 1). We included one carriage isolate, NZCM148, from the NZ96/294 household that was clearly distinct from the index patient isolate. It was included to test whether isolates within the same household share similarities regardless of sequence type. Maximum-likelihood phylogeny showed isolates separated into two major groups, corresponding to their sequence type (ST-42 versus ST-154). In addition, isolates from the same household clustered together, even within a shared sequence type (Fig. 1). Within the NZ96/294 household, carriage isolate NZCM148 belongs to a different clonal complex and did not cluster with the index disease-associated isolate.

**TABLE 1:** Epidemiological and molecular data associated with households in this study

Household	Isolate	Sequence type^a^	Age^b^ (yr)	Site^c^
NZ96/294	NZ96/294	ST-42	38	Blood
	NZCM149	ST-42		Throat
	NZCM148	ST-5159		Throat
NZ97/019	NZ97/019	ST-42	1	CSF
	NZCM162	ST-42		Throat
NZ97/021	NZ97/021	ST-42	3	CSF
	NZCM165	ST-42		Throat
NZ97/133	NZ97/133	ST-42	13	Blood
	NZCM132	ST-42		Throat
	NZCM133	ST-42		Throat
	NZCM134	ST-42		Throat
	NZCM135	ST-42		Throat
NZ97/192	NZ97/192	ST-154	15	CSF
	NZCM238	ST-154		Throat
	NZCM239	ST-154		Throat
	NZCM240	ST-154		Throat
NZ98/058	NZ98/058	ST-154	59	Blood
	NZCM246	ST-154		Throat
NZ98/074	NZ98/074	ST-154	18	Blood
	NZCM245	ST-154		Throat

a All isolates, apart from NZCM148, belong to the New Zealand epidemic serogroup B strain type: B:4:P1.7-2,4, ST41/44 clonal complex (lineage 3).

b Age of the index patient.

c Site where the meningococcal isolate was collected.

### Whole-genome sequencing identified variations among household isolates.

We extracted high-confidence variants between the household isolates (excluding the unrelated isolate NZCM148) and the New Zealand epidemic strain reference genome, NC_017518.1, and removed variants that were common to all isolates within a household group, variants within the *pilS* antigenic variation region, and predicted tandem repeats. Within the same household, the number of variants among isolates ranged from 9 to 210 (Table 2).

**TABLE 2:** Number of variants among the household isolates and their classification^a^

Household	No. of isolates in household	Total no. of variants (NC_017518.1)	No. of variants by type^b^:								
			I	U	D	S	In	De	M	F	Stop
NZ96/294	3	23	6	6	5	2	0	0	2	2	0
NZ97/019	2	210	28	20	19	99	0	2	37	5	0
NZ97/021	2	9	2	2	1	1	1	0	1	1	0
NZ97/133	5	51	20	4	4	6	2	2	5	8	0
NZ97/192	4	176	78	9	3	27	3	4	35	16	1
NZ98/058	2	97	37	1	9	17	3	1	21	7	1
NZ98/074	2	44	8	2	0	12	0	0	13	9	0

a The single nucleotide polymorphisms detected are listed, including information about the household group where they were identified, number of isolates in each household, and summary of the type of polymorphism. The divergent isolate NZCM148 has been omitted.

b Abbreviations: I, intergenic; U, upstream; D, downstream; S, synonymous; In, in-frame insertions; De, in-frame deletions; M, missense; F, frameshift; Stop, gained stop codon.

We annotated the variants according to their location and possible effect on coding sequences (CDSs) using SnpEff (19). For ease of annotation, variants were analyzed in “clumped” format, where variants close to each other were presented as one change. This method ensures that changes within a codon are annotated correctly. There were a total of 421 clumped variants; of these, 141 were classified as intergenic (defined as more than 100 bp away from an annotated transcript), 19 were located less than 100 bp downstream of an annotated transcript, and 40 were located less than 100 bp upstream of an annotated transcript. Within coding regions, 90 variants caused synonymous changes, 83 caused missense changes, and 38 were predicted to cause frameshifts in the coding region, with 10 in-frame insertions and 11 in-frame deletions (dels) also identified (summarized in Table S1 in the supplemental material). Many variants that were predicted to be upstream of coding sequences (17/40) or to cause frameshifts (27/38) were within homopolymeric and small-repeat sequences (Table 3). As variants within homopolymeric sequences are difficult to assess using whole-genome sequencing data, we confirmed some of these differences for the NZ97/192 family using Sanger sequencing (details in Table 3). Differences within homopolymeric sequences may indicate that these transcripts are under phase-variable regulation. Indeed, variants were found within genes that are known to be phase variable, including *lgtA*, *siaD*, *pilC*, *porA*, and those encoding opacity proteins. Interestingly, variants that were predicted to affect *siaD* translation were detected only in carriage isolates (3 isolates from two households). These three carriage isolates (NZCM238, NZCM240, and NZCM245) all had variants in single nucleotide repeat sequences that resulted in a translational frameshift of *siaD*. The presence of detectable capsule in these isolates was tested by slide agglutination. NZCM238 and NZCM245 were found to be nongroupable, confirming loss of the capsule, although results could not be determined for NZCM240, which exhibited extensive autoagglutination. Serogroup B capsule expression was confirmed by slide agglutination for the other isolates from the household, NZ97/192 and NZCM239. Other variants were detected in isolates from both patients and carriers. Six variants were found that restored open reading frames (ORFs) in orthologs of NMBNZ0533_0050 (*pilC1*), NMBNZ0533_0475 (*pilC1*), and NMBNZ0533_1834 (Dca), which are annotated as pseudogenes and are predicted to be degenerate in the reference genome.

10.1128/mSystems.00127-17.1 TABLE S1  Summary of variants between household isolates. This table lists all the identified genome variants, including the type of variant, position, affected locus, and predicted resulting amino acid sequence changes. The unrelated isolate NZCM148 was excluded from the table, as were variants common to all household isolates or those located in the pilin locus. DP < 10 indicates that fewer than 10 reads were obtained. Download TABLE S1, XLSX file, 0.1 MB. Copyright © 2017 Ren et al. 2017 Ren et al. This content is distributed under the terms of the Creative Commons Attribution 4.0 International license.

**TABLE 3:** Homopolymeric and small-repeat variants upstream and within annotated transcripts

Position	Reference sequence	Alternative sequence(s)	Location/effect	Locus	Change^e^
30823^a^	G(A)11C	G(A)10C; G(A)12C	Upstream	NMBNZ0533_0037	
45680^b^	C(G)9C	C(G)10C	Frameshift	NMBNZ0533_0050	
75462^a^	C(T)9G	C(T)10G	Frameshift	*siaD*	Lys142fs
75798^a^	T(G)7T	T(G)6T	Frameshift	*siaD*	Thr30fs
75872^a^	C(T)7A	C(T)6A	Frameshift	*siaD*	Ile5fs
338414^a^	A(G)9C	A(G)10C	Frameshift	NMBNZ0533_0325	Pro958fs
354493	CAG(10)	CGAGA(G)7A	Upstream	*fetA*	
388239^a^	C(G)9C	C(G)10C; C(G)11C	Frameshift	NMBNZ0533_0355	Pro96fs
424181^a^	C(G)8A	C(G)9A; C(G)7A	Frameshift	*lgtA*	Glu88fs
506007	G(C)12G	G(C)11G	Frameshift	NMBNZ0533_0475	
515698	C(G)11A	C(G)10A; C(G)12A; C(G)13A	Frameshift	NMBNZ0533_0485	Glu63fs
546125	C(G)9A	C(G)11A	Frameshift	NMBNZ0533_0508	Pro164fs
862493^c^	A(G)8C	A(G)7C	Upstream	*purC*	
931392	C(G)9A	C(G)10A; C(G)11A	Frameshift	NMBNZ0533_0877	Ter193fs
1119938^d^	C(G)11A	C(G)7A; C(G)10A; C(G)8A	Upstream	*opcA*	
1257866	C(G)9A	C(G)8A; C(G)7A; C(G)10A	Frameshift	NMBNZ0533_1221	Gly105fs; Phe106fs
1472851^a^	G(T)7G	G(T)8G	Frameshift	*msbB*	Lys143fs
1487249^c^	A(C)12G	A(C)11G; A(C)10G	Upstream	*porA*	
1529640	G(GAAGA)8G	G(GAAGA)7G; G(GAAGA)9G; G(GAAGA)10G	Upstream	NMBNZ0533_1444	
1598967	C(TGCT)7T	C(TGCT)5T	Frameshift	NMBNZ0533_1501	Glu4fs
1669031^c^	C(G)8T	C(G)9T	Frameshift	NMBNZ0533_1559	Val49fs
1721245	C(CTTCT)4C	C(CTTCT)7C; C(CTTCT)9C	Upstream	NMBNZ0533_1610	
1963892	C(A)5C	C(A)6C; (A)6C	Upstream	NMBNZ0533_1807	
1997634	A(C)7G	A(C)8G; A(C)9G; A(C)11G	Frameshift	NMBNZ0533_1834	Gly141fs

a Confirmed by Sanger sequencing within the NZ97/192 household isolates.

b Multiple PCR products.

c Mixed sequences detected by Sanger sequencing within the NZ97/192 household isolates.

d Not confirmed by Sanger sequencing within the NZ97/192 household isolates.

e fs, frameshift.

Of the 94 variants that were found to cause amino acid substitutions, insertions, or deletions, only 5 were predicted to alter protein function by PROVEAN (Protein Variant Effect Analyzer) (20, 21). These changes were found in NMBNZ0533_0397 (*lgtC*), NMBNZ0533_1030 (chloride transporter), NMNZ0533_1646 (*hmbR*), NMBNZ0533_1790 (*tbpA*), and NMNZ0533_1997 (citrate transporter family protein). *lgtC* encodes a glycosyltransferase that modifies lipo-oligosaccharide, and a Gly57 deletion mutation was found in all of the NZ97/192 household isolates. *hmbR* encodes a TonB-dependent hemoglobin receptor, a potential virulence factor involved in iron acquisition. The mutation Leu380dup was found in a carriage-associated isolate, NZCM135. *tbpA* has also been shown to be involved in iron acquisition. The mutation in *tbpA* (Gly795Ser) was found in NZCM134, a carriage-associated isolate. The NMBNZ0533_1030 gene encodes a chloride transporter protein, and a variant in this gene was found in several families and in both carriage- and disease-associated isolates. The NMBNZ0533_1997 gene encodes a citrate transporter protein, and the mutation Ala263del was identified in NZCM134, an isolate from a healthy carrier.

A large number of single nucleotide polymorphisms (SNPs) were identified in the invasive isolate NZ97/019 in an approximately 5-kb region (Table S1), suggesting the possibility of a recombination event with another isolate or species. To investigate the origin of this DNA region, the NZ97/019 draft genome was aligned with the reference genome, NZ-05/33. The sequence corresponding to bp 185000 to 190142 was extracted and searched against the PubMLST *Neisseria* database (22). For the six genes contained in this region, four from NZ97/019 had alleles that matched those found in an isolate of a related, carriage-associated resident of the nasopharynx, *Neisseria bergeri*. The top hits for the remaining two genes were also *N. bergeri-*associated alleles, although these were not identical. When the corresponding sequence from the reference genome, NZ-05/33, was queried in the database, all the alleles matched *N. meningitidis* isolates only. The most likely explanation for these observations is that the New Zealand epidemic strain acquired ~5 kb of DNA from a commensal neisserial strain while in the nasopharynx. *N. bergeri* is a newly proposed species, which was formerly considered a divergent strain of *N. polysaccharea* (23, 24). *N. polysaccharea* and *N. bergeri* are both commensal species, closely related to *N. meningitidis*, which reside in the nasopharyngeal mucosa. The exchange of DNA between *N. meningitidis* and other bacterial members of the nasopharyngeal microbiome has previously been documented (25, 26). We did not find any evidence that the associated throat isolate from the NZ97/019 household (NZCM162) had acquired DNA through recombination.

No consistent patterns were identified that could distinguish isolates from healthy carriers from those responsible for invasive disease, but many genomic differences were observed among isolates from the same household. Many of these genomic differences could result in altered transcription, with these differences underpinning different outcomes in the host. We therefore selected four isolates from the NZ97/192 household group for further phenotypic and transcriptome analyses.

### Household isolates do not differ significantly for cell association or invasion of bronchial epithelial cells.

**FIG 2:**
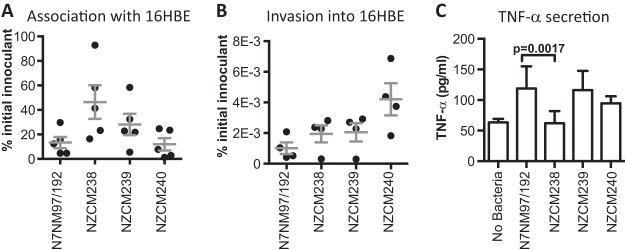
Isolates from the NZ97/192 household interact differently with host respiratory epithelial 16HBE cells. For each assay, all isolates were tested together and in biological replicates. Each assay was performed in triplicate at least twice. (A) Viable bacteria associated with the 16HBE cells (adhered and intracellular), expressed as a percentage of the initial inoculant. Horizontal bars indicate means, while error bars indicate standard deviations. (B) Viable bacteria present inside 16HBE cells after gentamicin treatment, expressed as a percentage of the initial inoculant. Horizontal bars indicate means, while error bars indicate standard deviations. (C) Concentration of TNF-α in the culture media of 16HBE cells and bacterial cocultures (picograms per milliliter, *n* = 6). The average value is plotted for each isolate, and error bars indicate standard deviations.

The ability to adhere to respiratory epithelial cells is essential for the initial colonization of the nasopharynx by meningococci. We measured the ability of our isolates to associate with 16HBE epithelial cells, an immortalized normal human bronchial epithelial cell line (27). Mid-log-phase bacteria were cocultured with cells, followed by washing and enumeration of cell-associated bacteria (adhered and intracellular). CFU recovered were normalized to the initial inoculant, also estimated by CFU counts. We found high variability in the ability of meningococcal isolates to adhere, but no statistically significant differences among the isolates. Numbers of cell-associated bacteria for the NZCM238 isolate were slightly higher than those for the other isolates, but this difference was not significant (Fig. 2A).

The ability to invade respiratory epithelial cells may render the bacteria invisible to immune surveillance and may also contribute to invasive disease development. We measured the isolates’ ability to invade 16HBE cells using a gentamicin protection assay and enumeration of intracellular bacteria. Invasion rates were low among all four isolates, and no statistically significant differences were seen. NZCM240 was slightly more invasive in this assay, but this did not reach statistical significance (Fig. 2B).

### Isolates differ in their ability to elicit TNF-α secretion in bronchial epithelial cells.

Tumor necrosis factor alpha (TNF-α) is a major proinflammatory cytokine induced during meningococcal infection, by monocytic cells (systemic) or epithelial or endothelial cells (locally). The levels of TNF-α in the blood positively correlate with disease severity, with higher levels of TNF-α corresponding to more severe disease (28). TNF-α expression in the mucosal epithelium in response to pathogenic neisseriae results in the efficient recruitment and activation of neutrophils (29, 30). We therefore infected synchronized 16HBE cultures with the different isolates and measured the amount of TNF-α in the culture medium after 24 h (Fig. 2C). The NZ97/192-infected 16HBE cells secreted the largest amount of TNF-α (118.8 ± 36 pg/ml [standard deviation {SD}], *n* = 6), though TNF-α levels in NZCM239-infected cultures (116.1 ± 31.5 pg/ml, SD, *n* = 5) were similar. The NZCM238-infected cultures had significantly smaller amounts of TNF-α (62.0 ± 20.0 pg/ml, SD, *n* = 6) than cultures infected with other isolates from within the household group. The level detected was similar to that observed in uninfected cultures (63.5 ± 5.7 pg/ml, *n* = 5). NZCM240 induced slightly lower levels than NZ97/192 and NZCM239, though this was not statistically significant. These assays demonstrated subtle phenotypic differences between these highly related isolates in their interaction with host epithelial cells.

### NZ97/192 household isolates have different transcription profiles in the presence of respiratory epithelial cells.

**FIG 3:**
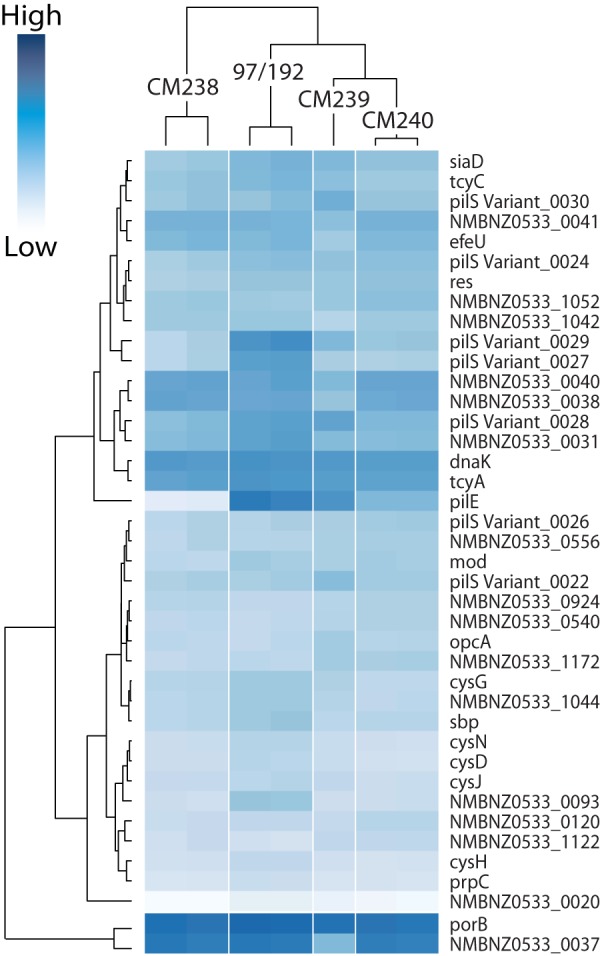
Heat map and dendrogram of differentially expressed transcripts among the NZ97/192 household isolates. Transcripts that were found to be significantly different by DESeq (−1 ≤ log_2_Δ ≥ 1; adjusted *P* < 0.05) are displayed. Dark blue represents high levels of expression. The dendrogram illustrates the clustering of replicate samples for a given isolate. For detailed transcript information, see Table S2.

We demonstrated that the isolates within the NZ97/192 household group interact differently with 16HBE cells, especially with the reduced ability of one isolate, NZCM238, to induce TNF-α secretion from 16HBE cells after infection. To understand how the transcriptional response of the isolates varies in response to 16HBE cells, we purified total RNAs from bacteria during mid-log phase while in coculture with 16HBE cells and compared their transcription profiles. For this analysis, we used the annotated RefSeq genome NMBNZ0533 as our reference. A total of 37 transcripts, excluding rRNA, were significantly different (adjusted *P* ≤ 0.05 as determined by DESeq) between NZ97/192 and one of the three carriage-associated isolates (Table S2) (31). Most of the genes were downregulated in the carriage-associated isolates relative to the disease-associated isolate. We also analyzed the replicates separately and found that most transcripts were confirmed in the replicate data set. A heat map of 37 transcripts with the largest differences (−log_2_ ≥ ±1, adjusted *P* ≤ 0.05) from both replicates is displayed in Fig. 3, which demonstrates that the replicates cluster together. In addition, NZCM238 clustered separately from the other isolates. One of the genes with the largest difference in expression is *pilE*, the expressed pilin gene, with almost no expression detected in NZCM238.

10.1128/mSystems.00127-17.2 TABLE S2  Summary of transcriptome sequencing gene expression differences between disease and carriage isolates. This table lists the genes whose expression differed significantly between the disease isolate (NZ97/192) and the associated carriage isolates from the same household. Gene ontology numbers from UniProt are listed, along with the calculated fold change and *P* values. Localization predictions by UniProt, PSORTb, and NeMeSys are also listed. Download TABLE S2, XLSX file, 0.1 MB. Copyright © 2017 Ren et al. 2017 Ren et al. This content is distributed under the terms of the Creative Commons Attribution 4.0 International license.

**FIG 4:**
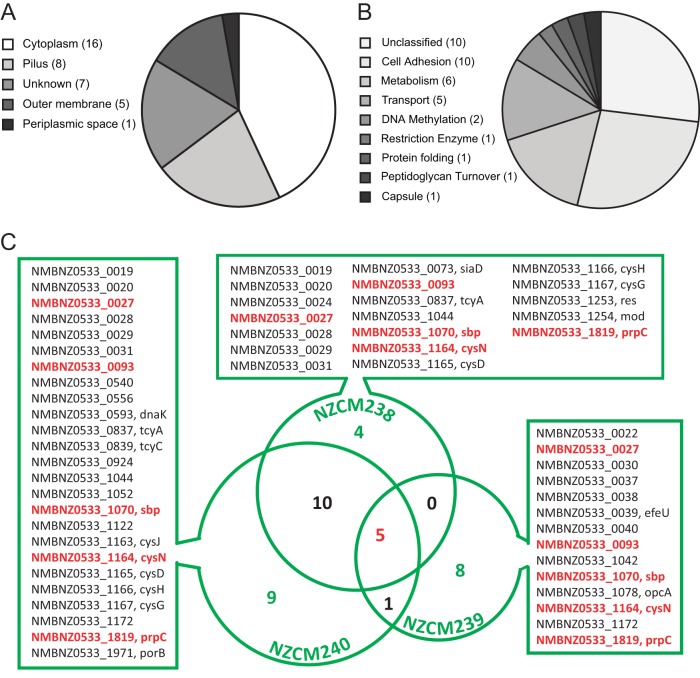
Predicted location and functional classification of differentially expressed transcripts. Transcripts were classified according to the subcellular location and predicted function of their encoded protein product, using the UniProt database. (A) Cellular location analysis shows that 16/37 transcripts were predicted to encode cytoplasmic proteins. (B) The function of 10 out of 37 transcript gene products could not be classified, either because they were absent from the UniProt database or because their function was unknown. Six transcripts encoded proteins predicted to be involved in metabolism, highlighting possible metabolic differences between disease- and carriage-associated isolates. (C) Venn diagram listing differentially expressed (log_2_Δ ≥ ±1; adjusted *P* < 0.05) transcripts between each carriage isolate and the disease isolate (NZ97/192). Five transcripts, highlighted in red, were differentially expressed in all three carriage isolates, relative to the invasive isolate. For detailed transcript classification, see Table S2.

We annotated the predicted gene products according to predicted cellular location and function using the UniProt database (32) (Fig. 4A and B). Additional localization information from PSORTb and NeMeSys is summarized in Table S2 (33, 34). One transcript was predicted to contain a tRNA (tRNA-Leu), while the remaining transcripts were predicted to encode intact or degenerate proteins. Subcellular location was not known for seven proteins, while six proteins were predicted to be located in either the periplasm or the outer membrane. Eight proteins were predicted to be associated with the pilus, and 15 proteins and the tRNA were predicted to be cytoplasmic. Functional prediction resulted in 10 genes with unclassified function; these included four transcripts missing from the UniProt database because they were not predicted to encode functional proteins (NMBNZ0533_0020, NMBNZ0533_0040, NMBNZ0533_1052, and NMBNZ0533_1044), one predicted to contain an RNA molecule (NMBNZ0533_1172, tRNA-Leu), and five with unknown function. UniProt predicted that six transcripts encode proteins involved in metabolic pathways, including four genes (*cysN*, *cysD*, *cysJ*, and *cysH*) that encode enzymes in the sulfate assimilation pathway. In addition to the genes located within the pilin variation locus, an additional transcript, a putative hemagglutinin, was also predicted to function in cell adhesion. Although OpcA was not predicted by UniProt to have a function in adhesion, we classified it as an adhesin, based on previous reports (35–38). Five proteins were predicted to function as transporters; the gene for one of these encodes a sulfate binding protein, which also functions in the sulfate assimilation pathway. PorB, a major outer membrane porin, which has been shown to translocate to the host cell mitochondrion, where it prevents apoptosis, was also classified as a transporter (39–41). Other transcripts were predicted to function in DNA methylation (*mod* and NMBNZ0533_0924), capsule biosynthesis (*siaD*), protein folding (*dnaK*), peptidoglycan turnover (MltA family protein), and restriction modification (*res*).

A Venn diagram and a list of the transcripts with significantly different expression (adjusted *P* ≤ 0.05) and their respective isolates are pictured in Fig. 4C. Four transcripts were uniquely different between NZ97/192 and NZCM238, while 8 transcripts were uniquely different between NZ97/192 and NZCM239 and 9 transcripts were uniquely different between NZ97/192 and NZCM240. Among these transcripts, 5 had decreased expression in all three carriage-associated isolates (red in Fig. 4C).

One of the 5 transcripts with decreased expression in all three carriage isolates was NMBNZ0533_0027, part of the *pilS* antigenic region. NMBNZ0533_0093 encodes a conserved hypothetical protein with a predicted outer membrane protein domain. This expression difference was confirmed by real-time quantitative PCR (qRT-PCR), where its expression was almost undetectable in the carriage isolates (>90% reduction, relative to invasive disease isolate, *P* < 0.0001, *n* = 9). Interestingly, 241 bp upstream of the predicted translation start site, there is a stretch of C’s that differed in number between NZ97/192 and all three isolates from healthy carriers. NZ97/192 contained 9 C’s, whereas NZCM238, NZCM239, and NZCM240 all had 10 C’s (confirmed by Sanger sequencing). This configuration is characteristic of genes that undergo phase variation, but it is not known whether this stretch of C’s contributes to transcriptional regulation. A second gene, NMBNZ0533_0037, may also undergo phase variation; a homopolymeric sequence of A’s is found 47 bp upstream of the translation start site. The NZ97/192, NZCM238, and NZCM240 isolates all have 10 A’s in this sequence, while NZCM239, which has significantly reduced expression of the gene, has 11 A’s. We were not able to confirm the difference in expression of NMBNZ0533_0037 in the isolates by qRT-PCR, due to extreme variability between the replicates. This may be due to stochastic switches occurring in the tested populations. NMBNZ0533_0037 has an ortholog in the *N. meningitidis* MC58 genome, NMB0032, which shares the same upstream sequence and homopolymeric A tract as NMBNZ0533_0037. NMB0032 has previously been identified as a likely “moderate” phase-variable gene candidate in the study carried out by Siena et al., although they did not find direct evidence of phase variation of this gene (42). However, we did observe differences in the numbers of A’s in our isolates (Table 3). NMBNZ0533_0093 has not, to our knowledge, been identified as a phase-variable gene elsewhere. Like many phase-variable genes, NMBNZ0533_0037 encodes a protein that is predicted to be membrane associated, and so it may be subject to selective pressure to evade an immune response (42, 43).

Three of the transcripts that are downregulated in all the carriage isolates (*sbp*, NMBNZ0533_1070; *cysN*, NMBNZ0533_1164; and *cysG*, NMBNZ0533_1167), encode proteins predicted to function in the sulfate assimilation pathway. *sbp* encodes a sulfate binding protein, *cysN* encodes the large subunit of a sulfate adenylyltransferase, and *cysG* encodes a siroheme synthase. For *sbp*, qRT-PCR confirmed reduced expression in NZCM238 and NZCM239 but not in NZCM240 (NZCM238, 55.6% ± 27.8% reduction, standard error of the mean [SEM], unpaired *t* test, *n* = 10, *P* = 0.023; NZCM239, 54.6% ± 16.2% reduction, SEM, unpaired *t* test, *n* = 10, *P* = 0.0075; NZCM240, 26.7% ± 22%, reduction, SEM, unpaired *t* test, *n* = 10, *P* = 0.105). However, qRT-PCR did not confirm the differences in transcription for *cysN* or for *cysG*. The high variability in the qRT-PCR experiment for *sbp* was mainly due to two sets of experiments where the level of expression was higher in the carriage isolates than in NZ97/192. The last transcript, *prpC* (NMBNZ0533_1819), encodes 2-methylcitrate synthase in the citrate synthesis pathway.

In addition to examining the expression differences, we also compared the sequences of the transcripts and predicted protein sequences from the isolates to identify variations, with potential functional consequences, by comparing predicted mutations to the Pfam database and protein orthologs. The only protein that was predicted to be functionally different between all three carriage-associated isolates, compared to NZ97/192, was a conserved hypothetical protein encoded by NMBNZ0533_0823. This gene encodes a small protein with a putative hemolysin domain. However, this predicted open reading frame (ORF) was of low quality and, like many of the transcripts predicted to have deleterious frameshift mutations, was not highly expressed under our assay conditions.

### Intracellular glutathione levels differ between throat and blood isolates following oxidative stress.

**FIG 5:**
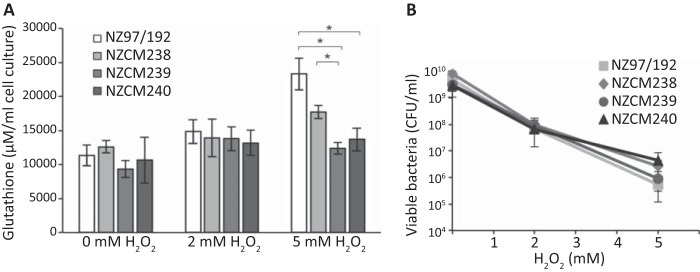
The invasive isolate has higher levels of intracellular glutathione following exposure to oxidative stress. (A) The decreased expression of sulfate assimilation genes in the carriage-associated isolates from the NZ97/192 household leads to lower levels of intracellular glutathione following exposure to 5 mM hydrogen peroxide. *, *P* < 0.05. (B) No significant difference was seen in survival rates in the presence of 2 or 5 mM hydrogen peroxide for 20 min. Error bars represent standard deviations.

The expression differed between NZ97/192 household isolates for multiple genes predicted to play a role in sulfate assimilation, including the sulfate binding protein, *sbp*, and a cystine ABC transporter, *tcyA*, as well as *cysG*, *cysH*, *cysJ*, and *cysN*. The products of the last four genes enable reduction of sulfate into hydrogen sulfide (33). The importance of sulfur metabolism for nasopharyngeal colonization has been previously noted for meningococci (44). Sulfur acquisition via the cysteine biosynthesis pathway is also essential for production of glutathione, a potentially important cellular antioxidant. Glutathione reduces oxidative stress by facilitating reduction of other proteins via cysteine thiol-disulfide exchange (45). To look for functional implications of this differential gene expression, we investigated levels of intracellular glutathione in isolates from the NZ97/192 household. Under normal conditions, glutathione levels were similar between all the isolates (Fig. 5A). However, following exposure to oxidative stress, induced by the addition of 5 mM hydrogen peroxide, significantly higher levels of intracellular glutathione were detected in NZ97/192 than in the carriage-associated isolates NZCM239 and NZCM240 (*P* < 0.05). Glutathione levels in NZ97/192 were also higher than those in NZCM238, although they did not reach statistical significance. Higher levels of glutathione are consistent with the higher expression of sulfur acquisition genes in NZ97/192. Under the conditions tested, however, we did not detect any difference in the ability of the isolates to survive hydrogen peroxide challenge for 20 min (Fig. 5B).

### T4P are associated with TNF-α secretion from respiratory epithelial cells.

**FIG 6:**
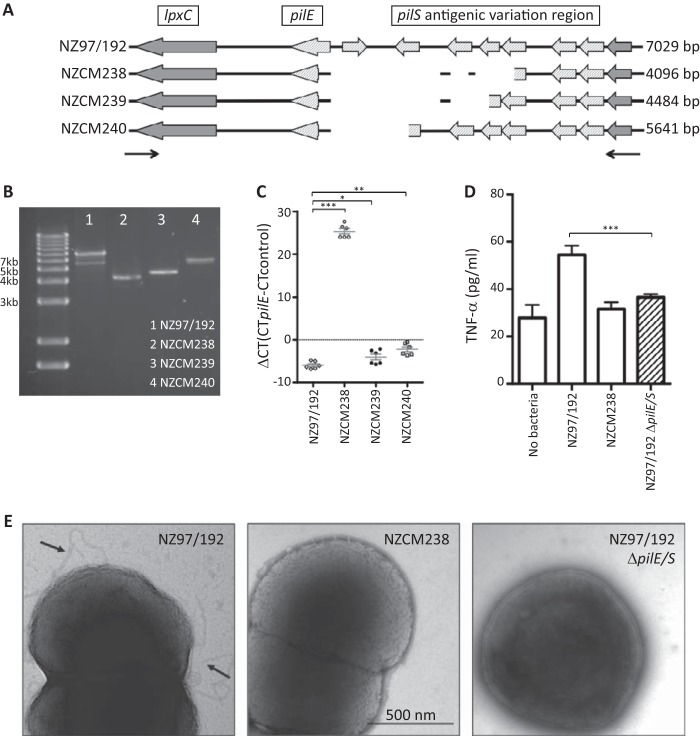
Lack of *pilE* expression in NZCM238 is associated with lower TNF-α secretion by 16HBE cells during coculture. (A) *De novo* assembly, annotation, and alignment of the *pilE-pilS* region of the isolates within the NZ97/192 household revealed a large amount of variability. Deletions of 2,933, 2,545, and 1,388 bp were detected in NZCM238, NZCM239, and NZCM240, respectively, compared to the index strain, NZ97/192. Block arrows depict predicted open reading frames. Small arrows below the drawing indicate primers used for genomic PCR. (B) Genomic PCR confirmed the length of the *pilE-pilS* region in each isolate, using primers indicated above. (C) Quantitative reverse transcriptase PCR (qRT-PCR) confirmation of reduced *pilE* expression in the carriage isolates, especially NZCM238. Threshold cycle (Δ*C*_*T*_) is presented in the graph, with higher Δ*C*_*T*_ corresponding to lower expression. There is no detectable expression in NZCM238 (*n* = 6), 71% ± 17.5% (SD, *n* = 6) reduction in NZCM239, and 94% ± 3% (SD, *n* = 6) reduction in NZCM240, relative to NZ97/192. (D) TNF-α secretion was significantly reduced when 16HBE cells were infected with NZCM238 or a *pilE-pilS* deletion mutant of NZ97/192 (NZ97192Δ*pilE*/*S*) compared with infection with wild-type NZ97/192 (*n* = 6). Error bars indicate standard deviations. *, *P* < 0.05; **, *P* < 0.001; ***, *P* < 0.0001. (E) Electron micrographs of NZ97/192 (left), NZCM238 (center), and the NZ97/192Δ*pilE/S* deletion strain. T4P are seen only with wild-type NZ97/192 (arrows).

*De novo* assemblies of NZ97/192, NZCM238, NZCM239, and NZCM240 predicted deletions within the *pilS* antigenic region in the three carriage-associated isolates compared to NZ97/192 (Fig. 6A), a finding consistent with reduced or absent transcription of *pilE* in the carriage isolates. To further characterize the pilin locus, we performed PCR against genomic DNA, using primers to transcripts surrounding the *pilS* region, and identified significant deletions in several isolates (Fig. 6B). PCR against genomic DNA from NZ97/192 yielded a 7-kb fragment, while NZCM238, NZCM239, and NZCM240 yielded fragment sizes of 4 kb, 4.5 kb, and 5.6 kb, respectively. The PCR fragment sizes were consistent with those predicted by genome assembly (Fig. 6A). As mentioned above, transcriptome analysis demonstrated that *pilE*, which encodes the major pilin subunit of type IV pili (T4P), and three other *pilS* open reading frames were downregulated in carriage-associated isolates. NZCM238 had the lowest expression of *pilE*, with a log_2_ fold change of −10.72. NZCM240 had the second lowest expression (log_2_ = −3.74), followed by NZCM239 (log_2_ = −1.24). We verified this difference using qRT-PCR, which showed no detectable expression (*n* = 6) in NZCM238, 71% (±17%, unpaired *t* test, *P* = 0.000164, *n* = 6) reduction in NZCM239, and 94% (±3%, unpaired *t* test, *P* < 0.0001, *n* = 6) reduction in NZCM240, all relative to NZ97/192 (Fig. 6C). T4P have been shown to induce TNF-α expression in endothelial cells (46); to investigate whether reduced *pilE* expression could explain the lower levels of TNF-α induction in epithelial cells, we constructed a pilin locus deletion. The entire *pilE* and *pilS* antigenic variation region from NZ97/192 was replaced with an antibiotic resistance marker. Using a TNF-α enzyme-linked immunosorbent assay (ELISA), we demonstrated that the NZ97/192 Δ*pilE/S* mutant was not able to elicit TNF-α from epithelial cells, suggesting that intact pili play a role in inducing TNF-α from respiratory epithelial cells (Fig. 6D). These data suggest that one explanation for the low TNF-α levels elicited by NZCM238 (Fig. 2C) is that this isolate lacks T4P. The absence of detectable T4P in NZCM238 and the NZ97/192 Δ*pilE/S* mutant was confirmed by transmission electron microscopy (Fig. 6E).

We were unable to complement the NZ97/192 Δ*pilE/S* mutant, which could not be transformed. However, deletions in the *pilE/S* locus in two other isolates described elsewhere (NZ98/254, a New Zealand epidemic disease-associated isolate, and NZCM107, an unrelated serogroup B carriage isolate [47]) also resulted in reduced TNF-α secretion. Additionally, the carriage-associated isolate NZCM246, also predicted to have a large deletion within the *pilE-pilS* region, confirmed by genomic PCR, showed a reduced ability to induce TNF-α relative to the household-associated disease isolate NZ98/058 (data not shown).

## DISCUSSION

In this study, we demonstrated evidence of meningococcal transmission within households, using whole-genome sequencing of closely related household contact isolates. Isolates from the same household were more closely related to each other, as evident by clustering on maximum-likelihood phylogeny, than to isolates of the same sequence type from other households (Fig. 1). To understand whether these genomic differences could impact how isolates interact with the host, we chose one household and examined phenotypic and gene expression differences among the isolates. We observed phenotypic and transcriptional differences (Fig. 2 and 3). We also linked genomic differences to expression and phenotype differences, using mutagenesis to demonstrate that the presence of pili correlates with TNF-α induction by meningococci in respiratory epithelial cells (Fig. 6). Analyzing the genetic and phenotypic differences of closely related disease- and carriage-associated meningococci may shed light on virulence mechanisms. All isolates studied here were originally collected for analysis of potential vaccine antigens, including the serogroup and PorA type, and so only a single colony was retained for long-term storage in the reference library. Because *N. meningitidis* is so highly heterogeneous, this study likely only captures a snapshot of the true diversity of the bacteria in both carriage and disease. Furthermore, because all household contacts of disease patients would have been treated chemoprophylactically for the disease, it is impossible to conclude that none of the healthy household contact carriers would have gone on to develop the invasive disease.

A similar approach has recently been published, involving deep sequencing of throat and invasive isolates from the same patients (43). This study, like ours, also identified phase variation switches and DNA rearrangements occurring in the pilin locus, although these rearrangements resulted in antigenic variation rather than loss of pili. This study concluded that most of the genetic variations that the authors detected resulted from stochastic processes, and they did not find evidence for within-host selection of invasive strains. Similarly, we did not identify any genetic signatures associated with isolates from the blood or CSF versus those from the throat.

It has long been known that household crowding is a risk factor for meningococcal disease (16–18). Our phylogenetic analysis supports transmission within households, although it is difficult to ascertain which isolate was the index isolate and how the transmission proceeded. In the NZ96/294 family, it may be argued that acquisition of the carriage isolate (NZCM149) and onset of the disease in a second household member occurred very close in time, as there are only 12 variants between those two isolates. The isolates in some households had more variants than others (Table 2), but this diversity did not seem to relate to patient age, nor did it correlate with whether the household contained members carrying a different strain. For example, there are 209 differences among the isolates within the NZ97/019 household, where the index patient was 1 year old and the household contact also carried the same strain, while in the NZ97/021 household, where the index patient was 3 years old and 1 of 2 household members sampled was carrying *N. lactamica*, only 10 differences were found. In the NZ97/019 isolate, we detected a likely recombination event, affecting a 5-kb region that likely originated from a related nasopharyngeal commensal species, *N. bergeri*, which could explain the large number of variants occurring in this household (23). We found that six genes in NZ97/019 expressed alleles associated with a carriage species. Interestingly, the recombination event was detected in only one invasive isolate, suggesting that the *N. bergeri*-derived alleles did not impact the virulence potential of the recipient isolate. All of the ST-42 and ST-154 isolates in our study had identical *mutS* and *mutL* alleles, suggesting that the differences in levels of variation between households were not due to differing hypermutator phenotypes. Another possible cause of high divergence in some families could be the length of carriage period in the index patient. In general, the incubation period for meningococcal disease is thought to be between 1 and 10 days. However, longer periods of carriage before disease development presumably can lead to higher diversity between the patient isolate and isolates from healthy household members, due to stochastic, recombination, and DNA rearrangement events.

High numbers of SNPs were found within households in our study, in contrast with a recent study of methicillin-resistant *Staphylococcus aureus* (MRSA) (48). When multiple household isolates of MRSA were analyzed by whole-genome sequencing, the mean number of SNPs found varied between 17.6 ± 35 and 12 ± 19. The much higher variability of *N. meningitidis* may be due to the natural competence of *N. meningitidis* and the documented ability of meningococci to take up DNA from the environment or from isolates during carriage (25, 26).

Many variants were found within mononucleotide runs and small-repeat regions among the household strains and were the result of slipped-strand mispairing, a major mechanism of phase variation of gene expression. Slipped-strand mispairing results in small heritable but reversible random changes that can result in “all-or-none” expression of regulated transcripts and proteins, when the homopolymeric tract occurs in the coding region, or more subtle modulations of gene expression, when the tract occurs in promoter regions (42). Genes that encode surface and exposed proteins of pathogenic bacteria often contain DNA repeats in intergenic or coding regions indicative of phase-variable gene regulation (43). The *siaD* gene, known to be phase variable, was found to be in an “off” state in three carriage-associated isolates, due to slipped-strand mispairing that resulted in a frameshift mutation; in contrast, *siaD* was predicted to be intact and expressed in all of the disease-associated isolates; these predictions were confirmed by slide agglutination tests (49). The presence of the capsule is one of the key features of invasive meningococcal strains. Changes in expression of other phase-variable genes were found in both disease- and carriage-associated isolates. The potential significance of these changes is difficult to assess. By comparing genome sequence and transcriptome expression, we have potentially identified two transcripts under phase-variable control, NMBNZ0533_0037 (a putative lipoprotein) and NMBNZ0533_0093 (a conserved hypothetical protein). The NMBNZ0533_0037 transcript is predicted to encode a protein that is located in the membrane or secreted, although the location of the predicted protein encoded by NMBNZ0533_0093 could not be determined.

In addition to surface molecules, predicted to interact with the host, our transcriptome analysis highlights the possible importance of metabolic differences in invasive disease development, in particular the sulfate assimilation pathway. Seven of the 37 most differentially expressed genes in the NZ97/192 household were predicted to participate in the sulfate assimilation pathway, which catalyzes the metabolism and uptake of sulfate from the environment. Assimilated sulfate is used to generate multiple important molecules, including cysteine, methionine, iron-sulfur proteins, and other sulfur-containing macromolecules. Several studies have shown the importance of sulfur-containing compounds for detoxification in *Salmonella*, *Bacillus*, and *Staphylococcus aureus* (50–52). Cysteine and sulfite reductases have been shown to be important for *Bordetella pertussis* and *Brucella melitensis* virulence (53, 54). In *Mycobacterium tuberculosis*, mutants in the pathway have attenuated virulence in mice (55). Little is known about the sulfate assimilation pathway in *N. meningitidis*, which efficiently acquires environmental sulfate and can grow on a variety of sulfur sources (56). It has been suggested that sulfite reduction is important for nasopharyngeal colonization, as genome comparisons have shown that both *N. meningitidis* and *N. lactamica*, which specialize in nasopharyngeal colonization, have intact *cysI-cysG* operons, while *Neisseria gonorrhoeae* does not (33). Our data suggest an additional role for the sulfate assimilation pathway in development of invasive disease. A recent study has shown that the sulfate assimilation pathway is upregulated when meningococci are cultured under low-cysteine conditions and under stress; similarly, several studies have found genes encoding sulfate transporter permeases to be among the first genes to be expressed upon adherence to the epithelium (13, 15, 57, 58). The CSF is a relatively nutrient-poor medium and is thought to have to low cysteine levels, although signature-tagged mutagenesis studies have revealed that sulfur acquisition genes are essential during growth on minimal medium but are dispensable in serum, suggesting that cysteine can be acquired from serum (59, 60). Invasive bacteria are likely to encounter reactive oxygen species, generated by immune cells. The generation of glutathione, via the cysteine biosynthesis pathway, is one means of protection of the cells from oxidative stress (44). Our data confirmed that higher intracellular glutathione levels, following oxidative stress, were found in an isolate with higher expression of sulfate assimilation enzymes, and elevated glutathione could be detected even after overnight incubation without oxidative stress. Although we did not detect any differences in the susceptibilities of the various isolates to oxidative stress, the impact of various levels of intracellular glutathione may be apparent under other conditions of stress. Our quantitative PCR analyses revealed that the expression of *sbp*, *cysN*, and *cysG* genes was more variable than the transcriptome sequencing (RNA-seq) analysis suggested; this may be due to the sensitivity of metabolic pathways to minor variations in culture conditions. Meningococcal sulfate assimilation enzymes, encoded by genes *cysI*, *cysJ*, *cysN*, *cysD*, and *cysH*, have high sequence identity to these enzymes in other bacteria. As for other bacteria, the genomic organization of genes encoding these enzymes suggests that they may be coregulated, as they are colinear with little spacing between each open reading frame. Our transcriptome analysis demonstrated that expression of these genes differed in similar direction and magnitude, a finding that supports coregulation. However, we did not identify putative transcription factors for their regulation. The role of sulfate metabolism in *N. meningitidis* carriage and disease is a subject of ongoing and future research.

T4P are an important virulence factor in *N. meningitidis*, participating in colonization and disease. Pili are major external structures that are exposed to the host immune system and therefore undergo extensive antigenic variation. Two classes of T4P have been described in meningococci. The New Zealand epidemic strain has class 1 pili, which are structurally similar to pili expressed in the related pathogen *N. gonorrhoeae*. The gonococcus has been shown to vary the amino acid composition of the major pilus protein, pilin (*pilE*), by a process of gene conversion (61, 62). The *pilE* locus can recombine with fragments of pilin coding sequence in the *pilS* region to alter the amino acid composition of the final protein. Variation of the expressed pilin gene and rearrangement of the pilin antigenic region have been demonstrated *in vivo*, in volunteer studies of *N. gonorrhoeae* infection, and, for *N. meningitidis*, during an accidental human passage and through comparison of throat and invasive isolates from the same patients (43, 63–65). Our study confirmed that pilin variation frequently occurs during carriage as well, as evidenced by the variations in the pilin antigenic region among multiple carriage isolates. We found that the degree of variation in the *pilS* region differed between household isolates. Within some household groups, there were almost no differences between disease- and carriage-associated isolates, while in others large deletions were found in carriage-associated isolates compared to their respective index disease isolate (e.g., NZCM238 and NZCM246). Genomic and transcriptome data suggest that NZCM238 lacks pili, an observation that was supported by electron microscopy (Fig. 6E) as well as our observation that NZCM238 could not be naturally transformed. Although pili have been frequently considered essential for colonization, it has been reported that *N. meningitidis* strains isolated from the nasopharynx are often nonpiliated, relying on alternative adhesins; under these circumstances, the opacity proteins (Opa and Opc) can mediate adhesion (66). Indeed, we did observe that the opacity proteins were expressed in our isolates and that there were not large differences in adherence to epithelial cells (data not shown and Fig. 2). In contrast, T4P have been shown to be essential during invasive infection of the host by meningococci. Specifically, type IV pili were shown to be essential to adhere to human endothelial cells lining dermal vessels, in a humanized mouse model, which had grafted human skin tissue (67). T4P also have been shown to induce microvasculature lesions and inflammation, which are responsible for the clinical symptoms of fulminant meningococcal disease (68). T4P have been shown to bind to CD147, a receptor of the immunoglobulin (Ig) superfamily, which is expressed at high levels on endothelial cells of the brain microvasculature (69). Although CD147 is also expressed in epithelial cells, additional mechanisms of adherence apparently enable nonpiliated carriage strains to persist in the host nasopharyngeal mucosa.

Using mutagenesis, we demonstrated that type VI pili are associated with the induction of TNF-α from respiratory epithelial cells. A similar observation has previously been made in endothelial cells, although these cells secreted TNF-α only when monocytic cells were also present (46). In contrast, no human TNF-α was detected during infection in a humanized mouse model (a SCID mouse with a human skin graft), suggesting that the transplanted vasculature, including endothelial and skin cells, is not the source of systemic TNF-α during meningococcal disease. Human interleukin-6 (IL-6) and IL-8, in contrast, were detected in this model system (67). A study in *N. gonorrhoeae* found that removal of the pili resulted in loss of TNF-α secretion by epithelial cells, although this was seen only for bacteria also lacking opacity proteins (Opa^−^) and hence was likely due to the reduced adherence of the Pil-Opa^−^ strain (70). However, our data suggest that the loss of TNF-α secretion, in our model system, is independent of adherence. Although NZCM238 was found to lack pili and did not elicit TNF-α secretion from 16HBE cells, this isolate adhered to 16HBE cells as well as, or better than, related piliated isolates. The differences reported in TNF-α secretion from epithelial cells could be due to the different bacterial species and host cell types tested.

Our findings suggest that pili are dispensable during meningococcal carriage and that they may even be detrimental to colonizing bacteria in the nasopharynx, as their presence could potentially trigger a localized proinflammatory response by the host, resulting in an influx of neutrophils and removal before transmission to a new host can occur. Whether meningococci actively alter the host immune response to facilitate long-term carriage, through either suppression of local cytokine expression or other means, remains an unanswered question. Also unknown is whether local proinflammatory cytokine production plays a role in the development of invasive disease, the process by which virulent strains access deeper tissues following a breach of the epithelial layer in the throat.

The fact that the T4P of *Neisseria meningitidis* are dispensable for colonization of mucosal surfaces highlights a key difference between *N. meningitidis* and the related pathogen *N. gonorrhoeae*, which uses pili to adhere to mucosal surfaces, where it elicits a strong local inflammatory response. This response leads to an influx of neutrophils, which do not control the infection; viable gonococci have frequently been observed inside neutrophils, where they delay fusion with granules, interfere with the respiratory burst, and delay apoptosis (71–74). The means by which carriage *N. meningitidis* suppresses a local inflammatory response, allowing it to persist in a healthy host for long periods, remain unknown. Although tissue culture cells only recapitulate some of the interactions between *N. meningitidis* and the host, they are a useful tool, as *N. meningitidis* is human restricted, and animal models for nasopharyngeal colonization have not yet been developed. The interaction is likely to be influenced by other cell types, including innate immune cells and other microbes of the normal microflora; much remains to be discovered about the complex interaction between host and microbe.

## MATERIALS AND METHODS

### Bacterial strains and growth conditions.

*N. meningitidis* isolates were collected as described during a household contact study carried out in Auckland in the late 1990s (12). Isolates were maintained by the Meningococcal Reference Laboratory (MRL) at the Institute of Environmental Science and Research (ESR), as part of the surveillance of meningococcal disease in New Zealand on behalf of the Ministry of Health. The carriage isolates described in the present study (designated NZCM) were collected from healthy patient contacts by peroral nasopharyngeal swab (12). Although three colonies were collected from each individual, where possible, the studies described here were performed on bacteria derived from single colonies that were stored at −80°C long-term (12). Isolates from the index patient were cultured from blood or CSF samples. All isolates were routinely typed using serological and sequencing methods to determine serogroup and serosubtype (*porA* allele); in addition, selected strains were further analyzed, with the serotype (*porB* allele) determined by serology and sequence type (ST) determined by multilocus sequence typing (MLST) as described elsewhere (75–77). Serogrouping was typically carried out using slide agglutination; where these results were ambiguous, or where the isolate was autoagglutinating, PCR was used to determine serogroup. The subtype was determined by whole-cell enzyme immunoassay; isolates that were not subtypeable were subjected to *porA*-PCR analysis. Slide agglutination was also carried out for isolates in this study that had undergone phase variation of the *siaD* gene, using antiserum to the serogroup B New Zealand epidemic strain type, a gift from ESR. All isolates in the MRL were immediately frozen in Trypticase soy broth with 15% glycerol at −80°C following minimal laboratory passage. Frozen working stocks of frequently accessed isolates are maintained to prevent repeated freeze-thaw cycles. *N. meningitidis* was grown on Columbia blood agar (CBA) plates (Fort Richard Laboratories, Auckland, New Zealand), for routine passage, or on BBL brain heart infusion (BHI) agar (Oxoid) plates, supplemented with kanamycin (50 µg/ml) where required, at 36°C in a humidified 5% CO_2_ incubator. *Escherichia coli* strain DH5α (subcloning efficiency) or Top10 cells (both from Life Technologies) were used for DNA manipulation and were grown on Luria-Bertani agar plates supplemented with kanamycin (50 µg/ml) or ampicillin (100 µg/ml).

### Genomic DNA purification.

Genomic DNA was purified from meningococci with the Gentra Puregene Yeast/Bact. kit (Qiagen, USA) according to the manufacturer’s instructions, with the following changes. Bacteria, grown on CBA plates overnight, were scraped from the plate (~10 µl), resuspended in 300 µl of lysis buffer, and incubated at 56°C for 1 h to kill meningococci, after which the remaining steps were followed. DNA concentration and quality were assessed by electrophoresis, 260/280 ratio, and the Quant-iT PicoGreen double-stranded DNA (dsDNA) assay kit (Thermo Fisher, USA).

### Whole-genome sequencing, assembly, and variation analysis.

Isolates were sequenced by New Zealand Genomics Limited (NZGL). NZ97/192 and NZCM238 samples were prepared using the Nextera DNA library format, and NZCM239 and NZCM240 samples were prepared using the TruSeq library format, while all other isolates were prepared with TruSeq Nano libraries (Illumina, USA). All samples were sequenced by Illumina MiSeq using paired-end sequencing, 2 by 150 bp for NZ97/192 and NZCM238 and 2 by 250 bp for all other isolates. Adaptor trimmed reads were quality trimmed using Trimmomatic-0.32 at a Phred quality greater than 20 and a minimum length of 100 bp (78). Paired reads were aligned to RefSeq genome NC_017518.1 (*Neisseria meningitidis* NZ-05/33), and variants, single nucleotide variants (SNPs) and small insertions and deletions (indels), were identified using Freebayes 1.0 and GATK 3.3.0 UnifiedGenotyper tool with ploidy set to 1 (79, 80). For Freebayes workflow, reads were aligned using Bowtie 2.1.0, with settings to allow for local alignments (options: -nondeterministic, -local) and converted to sorted and indexed BAM files using SAMtools-0.1.19-44428cd (options: -S -b -q 20) (81, 82). For GATK workflow, reads were aligned using bwa mem and converted using Picard-tools-1.119 (Broad Institute). GATK variants were filtered by GATK hardfilter setting. Freebayes variants were filtered by vcffilter within the vcflib toolkit (83). Variants were kept if they were found by both Freebayes and UnifiedGenotyper and had a quality of >30, an alternate frequency of >70%, a depth of >10, and at least 1 alternate read in forward and reverse direction. Variant annotation and effects were predicted by SnpEff (19). Common variations among groups of isolates were obtained using the isec function in bcftools (84). Select variants were confirmed by PCR and Sanger sequencing. *De novo* assemblies were carried out using SPAdes 3.5.0 (85), and contigs greater than 500 bp were kept and annotated with Prokka 1.12 (86). Functional change prediction was determined using the PROVEAN protein webtool (http://provean.jcvi.org/seq_submit.php). Predicted amino acid variants were compared to the NZ-05/33 reference protein sequence and were determined to be deleterious if the PROVEAN score was less than −2.5.

### Analysis of relatedness among household isolates.

Using the published New Zealand epidemic strain type (RefSeq genome NC_017518.1; *N. meningitidis* NZ-05/33, New Zealand epidemic strain type, 2005) as a reference genome, we identified genetic differences (deletions [dels], indels, and SNPs) between the isolates and the reference strain (87, 88). Core SNPs were identified using Snippy and Snippy-core (v 3.1) (87). Maximum-likelihood phylogeny was generated using PhyML with the HKY substitution model with bootstrapping (200×) (89, 90). To investigate the origin of a highly variable 5-kb region, the draft genome from isolate NZ97/019 was aligned to the reference genome, NZ-05/33, and the genomic region corresponding to NZ-05/33 bp 185000 to 190142 was extracted and searched against the *Neisseria* multilocus sequence typing website (https://pubmlst.org/neisseria/) (22). The resulting alleles were then used to identify the species of isolates that they were associated with.

### Cell culture and infection.

Bronchial respiratory epithelial cells (16HBE14o-, abbreviated as 16HBE [27]) were routinely cultured in M199 medium supplemented with 10% inactivated fetal calf serum (FCS). For infection experiments, 16HBE cells were suspended at 6 × 10^4^ cells/ml and cultured in 6- or 24-well plates until confluent. Prior to initiating infection, cells were washed with phosphate-buffered saline (PBS) and incubated in serum-free M199 for 16 to 24 h.

### Enumeration of cell-associated and intracellular bacteria.

The number of 16HBE cell-associated bacteria (i.e., adherent and intracellular) was quantified using a method previously described (47). Briefly, confluent, serum-starved cells in 6-well plates were infected with *N. meningitidis* at a multiplicity of infection (MOI) of ~10. After 4 h, cells were washed three times with warm PBS to remove non-cell-associated bacteria and lysed with 1% saponin in PBS. Dilutions of the lysate were plated out; input bacteria were also enumerated by plating a series of dilutions. To determine the number of intracellular bacteria, the same method was used, but 100 µg/ml gentamicin was added for 1 h and then removed by washing prior to addition of saponin, to kill extracellular bacteria. All isolates were assayed in parallel with three replicates per isolate. Experiments were performed at least twice. Statistical significance was tested using an unpaired *t* test.

### TNF-α secretion from 16HBE cells.

Confluent, serum-starved 16HBE cells in 24-well plates were infected with *N. meningitidis* at an MOI of ~10. After 24 h, cell culture medium was collected and centrifuged to remove bacteria. Aliquots of cleared medium were stored at −80°C until ready for use. A commercially available ELISA kit was used to determine TNF-α levels, according to the manufacturer’s instructions (R&D Systems, USA). All isolates were assayed in parallel with three replicates per isolate. Experiments were performed at least twice. Statistical significance was tested using an unpaired *t* test.

### RNA-seq sample preparation.

Total RNA was isolated from mid-log-phase *N. meningitidis* bacteria that were exposed to 16HBE cells. *N. meningitidis was* scraped from fresh plates and resuspended at an optical density at 600 nm (OD_600_) of 0.25 in M199 medium supplemented with 10% FCS. This bacterial suspension (1 ml) was added to 24-well plates with confluent 16HBE cells, to activate expression of genes induced by host cell factors. After 2 h, the bacterial suspension (900 µl) was combined with 100 µl of stop solution, 5% buffer-equilibrated phenol (pH 7.4) in ethanol. Total RNA was then purified essentially as described by Gaynor et al. (91). Briefly, after the bacterial suspension was combined with stop solution, the cells were collected by centrifugation for 5 min at room temperature. The pellets were frozen at −80°C until needed and then briefly thawed at room temperature and resuspended in 50 µl of 0.4-mg/ml lysozyme (Sigma) in 10 mM Tris, pH 8.0, 1 mM EDTA, and incubated for 5 min. Cells were lysed by adding 950 µl of Trizol reagent (Thermo Fisher, USA) and vortexing, followed by the addition of 200 µl of chloroform. After centrifugation at 14,000 × *g* for 15 min, the top phase was combined, in a new tube, with an equivalent volume of 70% ethanol. This solution was then applied to an RNeasy Mini column (Qiagen) and washed according to the manufacturer’s instructions. Samples were treated with an on-column RNase-free DNase kit (Qiagen). RNA concentration and quality were assessed with a 2100 Bioanalyzer instrument (Agilent Technologies); only samples with an RNA integrity number of >8 were used for subsequent steps. rRNA was removed using a MICROBExpress bacterial mRNA enrichment kit (Thermo Fisher, USA), according to the manufacturer’s instructions. The Bioanalyzer was used again to check the RNA following the mRNA enrichment step. Double-stranded cDNA was generated from 3 µg of enriched bacterial mRNA using the SuperScript double-stranded cDNA synthesis kit (Thermo Fisher, USA). The quantity of cDNA was determined with the Quant-iT PicoGreen dsDNA assay kit (Thermo Fisher, USA), while the quality was checked by PCR of a housekeeping meningococcal gene (*aroE*). Double-stranded cDNA (>700 ng/sample) was sequenced by Ambry Genetics (CA, USA). TruSeq 2- by 100-bp paired-end libraries were generated, indexed, and sequenced on a HiSeq lane, producing 26 GB of compressed sequence data.

### Analysis of transcriptome sequencing data.

Bowtie2 version 2.1.0 (82) was used for mapping quality-trimmed sequences to targets. A Bowtie2 index was built for Silva large-subunit and small-subunit rRNA sequences (version r111). This index was used to prefilter reads with Bowtie2 to exclude rRNA sequences. The NZ-05/33 genome was downloaded from NCBI (http://www.ncbi.nlm.nih.gov/nuccore/385856165) and extended by 209 bp to the end of the sequence to account for circular sequence. The NZ-05/33 transcriptome GTF file was downloaded from Ensembl (http://bacteria.ensembl.org/info/data/ftp/index.html) (92), converted to gff3 format, and altered to make identifiers (IDs) unique. Transcripts were extracted and converted to FASTA format using a custom script, extending 51 bp from the gene limits. A local Bowtie2 mapping (-k 2 --local --no-unal --no-mixed) was carried out for non-rRNA sequence against the extended *N. meningitidis* NZ-05/33 genome for all samples. Raw counts for each Bowtie/transcript mapping were generated using SAMtools. DESeq (31) was used to normalize the count data and produce differential expression probabilities, using an “all-versus-all” comparison of each isolate against each other isolate. Two sets of differentially expressed genes (for any comparison) were produced: a strict set with a DESeq adjusted probability of <0.05 and a relaxed set with either a DESeq adjusted probability of <0.1 or a fold change of >2×. DESeq was used to create a variance-stabilized transformation of the transcript counts, and heat maps of differentially expressed genes were created from these normalized expression values. Gene ontology assessments were performed by UniProt (32). Predicted subcellular localization was determined using PSORTb analysis, and functional predictions were checked with NeMeSys (https://www.genoscope.cns.fr/agc/microscope/collabprojects/nemesys.php) (33, 34). The Venn diagram was generated with BioVenn (93).

### Analysis of transcript variants.

The raw fastq files from RNA-seq were cleaned for low-quality segments, stretches of N’s, and inclusion of adaptor sequence. Overlapping read pairs were collapsed into single reads, using AdapterRemoval, v. 1.5.4 (94). The cleaned-up reads were then mapped to the reference genome (http://www.ncbi.nlm.nih.gov/nuccore/CP002424) using Bowtie2 with default parameters (82). The BAM files were processed using SAMtools (-q 25 -Q 0) to generate mpileup output (81). This output was used to create high-confidence genotypes for each strain using SNPest v. 1.0 (minimum depth of 10 reads, minimum posterior probability of 0.999, minimum support for indel of 90%) (95). The reference EMBL file was processed using an in-house script to extract all coding sequence (CDS) regions, including protein_ID, start and end coordinates, strand, DNA sequence, and the encoded protein sequence. A custom program that reads the list of CDS regions and a list of high-quality genotypes (SNPs and indels) was implemented. If one or more predicted mutations were in a CDS, the corresponding DNA sequence was changed, and the mutated protein sequence was generated. A new FASTA file was generated for each strain containing every mutated protein relative to the reference. These files were then compared to the reference proteins. Orthologous proteins were identified by identifying the best reciprocal matches in a pairwise proteome comparison, performed with phmmer from the HMMER3.0 package, and then the bit scores for individual domains in NZ97/192 were subtracted from the NZCM238 scores to produce a delta-bit score (DBS) (96–98). Genes with DBS values in the top 1% were flagged as potentially functionally important and further inspected for mapping error.

### Sanger sequencing of selected variants.

Selected variants were confirmed by PCR and Sanger sequencing. PCR assays were carried out with platinum *Taq* high-fidelity DNA polymerase (Thermo Fisher, USA) and purified genomic DNA. Primers are listed in Table S4 in the supplemental material.

### qRT-PCR.

Quantitative reverse transcriptase PCR (qRT-PCR) primers and probes were designed with the RealTimeDesign software (Biosearch Technologies, Inc.) and were generated by Life Technologies. Real-time primers were labeled at the 5′ end with 6-FAM (6-fluorescein amidite) fluorescent dye and were coupled with BHQ-1 quencher (black hole quencher 1). PCR specificity was determined by agarose gel electrophoresis and sequencing of products. For simplicity, all PCR assays were carried out using 250 nM probe and 200 nM primer (Table S3), using TaqMan universal master mix II with uracil-*N*-glycosylase (UNG; Thermo Fisher, USA). The following cycling program was used: 52°C for 2 min (1 cycle), 95°C for 10 min (1 cycle), and 95°C for 10 s and 58°C for 1 min (45 cycles). Reaction efficiency was determined using a standard curve with different concentrations of genomic DNA. The meningococcal genes *aroE* and *ctrA* were used as controls as RNA-seq analysis indicated that their expression did not significantly differ among the isolates. All assays were performed in parallel with three technical replicates per sample. Statistical significance was determined using an unpaired *t* test.

10.1128/mSystems.00127-17.3 TABLE S3  Primers for qRT-PCR. This table lists all qRT-PCR primers and probes, including sequence and fluorescent probe modification, used in this study. Download TABLE S3, DOCX file, 0.1 MB. Copyright © 2017 Ren et al. 2017 Ren et al. This content is distributed under the terms of the Creative Commons Attribution 4.0 International license.

10.1128/mSystems.00127-17.4 TABLE S4  Primers for PCR and Sanger sequencing. This table lists the primers used for PCR and Sanger sequencing and identifies the targeted locus, primer name, and sequence. Download TABLE S4, DOCX file, 0.02 MB. Copyright © 2017 Ren et al. 2017 Ren et al. This content is distributed under the terms of the Creative Commons Attribution 4.0 International license.

### Hydrogen peroxide sensitivity assay and measurement of intracellular glutathione levels.

Fresh cultures of *N. meningitidis* were scraped from CBA plates and resuspended in brain heart infusion (BHI) broth (Oxoid). Bacteria were incubated at 37°C in a 5% CO_2_ incubator until the OD_600_ reached 0.6. Cultures were split into three aliquots of 1 ml each. Hydrogen peroxide (Sigma-Aldrich) was added to two of the cultures, at 2 and 5 mM final concentrations, while the third culture was not exposed. Cultures were incubated at 37°C in a 5% CO_2_ incubator for 20 min. To remove hydrogen peroxide, the bacteria were pelleted at 400 × *g* for 10 min. The supernatant was discarded, and the pellets were resuspended in 1 ml BHI broth. Viable bacteria were enumerated by plating dilutions onto CBA plates. Following overnight growth, bacteria were scraped from the CBA plates (separate from those used to count colonies) and resuspended in PBS at an OD_600_ of 1.0. Bacteria were pelleted by centrifugation at 1,610 × *g* for 10 min, and the supernatant was discarded. The bacterial pellet was deproteinized by suspension in 3 volumes of 5% 5-sulfosalicylic acid solution and then lysed with two freeze-thaw cycles, alternating between liquid nitrogen and a 37°C water bath. Samples were placed at 4°C for 5 min and then centrifuged for 10 min at top speed in a microcentrifuge. Glutathione concentrations were assayed using a glutathione assay kit (Sigma-Aldrich). Experiments were performed three times, in triplicate.

### Analysis of pilin locus.

The pilus genomic region from NZ97/192, encoding NMBNZ0533_0019 (the major pilin PilE) and the silent PilS antigenic variation region, was deleted and replaced by the AphA3 kanamycin resistance cassette by natural recombination, essentially as described previously but in a different host isolate (47). PCR of the *pilE*/*pilS* locus was carried out using primers pilEF1-HindIII and pilER1-BamHI (47).

### Electron microscopy of *Neisseria meningitidis*.

Bacteria and type 4 pili were visualized by transmission electron microscopy. Fresh cultures of bacteria on CBA plates were scraped into a 2% glutaraldehyde solution in PBS. The bacteria were then processed for negative staining essentially as described by Burghardt and Droleskey (99) and imaged at the Otago Centre for Electron Microscopy.

### Accession number(s).

The whole-genome sequencing and transcriptome sequencing reads are available through the EMBL Nucleotide Sequence Database (ENA; https://www.ebi.ac.uk/ena) under study accession numbers PRJEB23076 and PRJEB23077, respectively.
