# Supplementary material for: Genomic, Transcriptomic, and Phenotypic Analyses of Neisseria meningitidis Isolates from Disease Patients and Their Household Contacts
Source: mSystems. 2017 Nov 14;2(6):e00127-17. doi: 10.1128/mSystems.00127-17 (PMC5686521; doi:10.1128/mSystems.00127-17)
Supplement: TABLE S3 [file sys006172149st3.docx]

| Oligo Name | Sequence (5’-3’) |
| --- | --- |
| aroE-P-1 | FAM-CCAACCGTACCCATGCCAAAGCC-BHQ1 |
| aroE-F1 | GTCCTGCCCGTATCGTCAT |
| aroE-R1 | TCGGGACGGCTTCAATGC |
| ctrA-P-1 | FAM-TCCTTCATCAGGCCCCAGCG-BHQ1 |
| ctrA-F1 | TTGTGTGGAAGTTTAATTGTAGGATGC |
| ctrA-R1 | TCAGATTGTTGCCCTAAAGAGACA |
| cysG-P1 | FAM-CCGTCTGAAACCAGCGCGTCGT-BHQ1 |
| cysG-F1 | GCGGACGAGGGACAGTATG |
| cysG-R1 | GGCTGCTGACGCTCAAAG |
| cysN-P1 | FAM-TTCATCATCGCCGACACGCC-BHQ1 |
| cysN-F1 | CCATATTGCGCGTGTATTGT |
| cysN-R1 | CATTACGATCGATGTGGCAT |
| NM0037-P1 | FAM-TCGAACGCAGGCAGTATGTCA-BHQ1 |
| NM0037-F1 | ACGACGGGAAAGAGCTGTATG |
| NM0037-R1 | TCATCGCCGCATCGGTTT |
| NM0093-P1 | FAM- AAGCCTGTACCGCCAAACTGCA-BHQ-1 |
| NM0093-RF1 | CAAGCCGGTATCGGAAAGCAT |
| NM0093-RR1 | TGGCTGCCGACTGTTTCTG |
| NMsbp-P1 | FAM-ATCGGCTTGAAGGCCGTTGG-BHQ1 |
| NMsbp-RF1 | AGGATTGGTTCATGGTTACGAC |
| NMsbp-RR1 | AGCAAACAGGCATTATCCGTAG |
| pilE-P-1 | FAM-TACACAGCCCGCGCACAAGTTT-BHQ1 |
| pilE-F1 | TCGCCCTTCCTGCTTATCAAG |
| pilE-R1 | CGGGCCATATGCCGTGATTC |

FAM: 6-fluorescein amidite

BHQ1: Black hole quencher
