# Supplementary material for: Genomic, Transcriptomic, and Phenotypic Analyses of Neisseria meningitidis Isolates from Disease Patients and Their Household Contacts
Source: mSystems. 2017 Nov 14;2(6):e00127-17. doi: 10.1128/mSystems.00127-17 (PMC5686521; doi:10.1128/mSystems.00127-17)
Supplement: TABLE S4 [file sys006172149st4.docx]

| Position (NC_017518) | Oligo Name* | Sequence (5’ -3’) |
| --- | --- | --- |
| 45680 and 45695 | NM97192_snp21-F | AGCTAAGTTCAGGACAGCCG |
|  | NM97192_snp21-R | TTACCAAGCTAGACGGCACG |
|  | NM97192_snp21S-F | AAACCGGAAACCTTGCCGTA |
|  | NM97192_snp21S-R | TTTCAACCAGCAATTCGGCG |
| 75462 and 75798, 75872 | NM97192_snp20-F | TGAAACATTATTTGGTAAGGTCGC |
|  | NM97192_snp20-R | GCATATGTTCCAGTCCCTTCAT |
|  | NM97192_snp20S-R | TGGCCTGTAAAAGACAGCAT |
| 90716 | NM97192_snp19-F | GGCGTTGGGTTTGAACAAGG |
|  | NM97192_snp19-R | TTGCCGTTTGTAGCCTTGGA |
|  | NM97192_snp19S-F | GGCGTTGGGTTTGAACAAGG |
|  | NM97192_snp19S-R | TTCGGCGATATGGCGATTCA |
| 98383 | NM0093_B_F1 | gcgggatccGCTATTCCATCGATTAGGGGCA |
|  | NM0093_B_R2 | gcgggatccATCGTTACGCCTGAGTCTTTGT |
|  | NM0093_seqF1 | CCGTCTTTTCCTGCGTTTCCTA |
|  | NM0093_seqR1 | GGTAAGTAAAAGAACCGTCGGC |
| 338414 | NM97192_snp3-F | CCTGTCTGCTGATGTCGGTT |
|  | NM97192_snp3-R | AAGGCAAAACACCGGAATGC |
|  | NM97192_snp3S-F | ATCGGCATAGCCGTTTTGGA |
|  | NM97192_snp3S-R | GCTTGACGGCGCACAAATTA |
| 388239 | NM97192_snp4-F | GATTCGCCTGTGTCGACGAT |
|  | NM97192_snp4-R | CAGCAACAGCAGAGCAACAA |
| 424181 | NM97192_snp6-F | TCCCGCACCGTATGTTTTCG |
|  | NM97192_snp6-R | TGTATGGGGTTGCCGAAAGG |
|  | NM97192_snp6S-F | GATTTATTGACGGGGCAGGC |
|  | NM97192_snp6S-R | TTCCATCTCGCCCACGATTT |
| 704695 | NM97192_snp7-F | TTCGTTCAGCCCCTGTTTGA |
|  | NM97192_snp7-R | TGACACACCACGACCTGAAG |
|  | NM97192_snp7S-F | CATCGGAGCCGTTATGGACA |
|  | NM97192_snp7S-R | CAAAAGGGGCTGATGTTCGG |
| 740488 | feABC_F1 | TTAAGGAACGGATAAAGGCATC |
|  | feABC_R1 | TCATCCTACTGATTGCCCTACC |
|  | feABC_seqf1 | TACTGATTGCCCTACCGCTTAC |
|  | feABC_seqR1 | ACTGTCCCCCAAAAGCCTTC |
| 862493 | NM97192_snp9-F | GTCGACGGCAGTATAGGCAG |
|  | NM97192_snp9-R | TCTGCGTCAGAATCTCCCCT |
|  | NM97192_snp9S-F | CGCCGACTTCGGCTAGAATA |
|  | NM97192_snp9S-R | AGCGACCATCAGCATACGTT |
| 1119938 | opcA_f1 | GGGTTGATATACCAGCCGTTAC |
|  | opcA_R1 | ATTGTTGTCGCTTCGGATAAAT |
|  | opca_seqF1 | AGGGCGATCATGGCATATGTA |
|  | opca_seqR1 | AAATTTTTACCGTGTTGGGTTC |
| 1119938 | NM97192_snp11-F | GGGTGTAGGTGTAGCCTGTG |
|  | NM97192_snp11-R | TAACCGAAACCGGACGAACC |
|  | NM97192_snp11S-F | TTTGAAGCTCTTGTGCAGCG |
|  | NM97192_snp11S-R | GGCGGCGGGTTTCTGT |
| 1472851 | EC231_f1 | CGGGTATCACTTTTGCATTTG |
|  | EC231_R1 | CATTTGTATTTTGCCGTCTGAA |
|  | EC231_seqF1 | GTTGCGTCCGAAATCCTGATC |
|  | EC231_seqR1 | GGCGAAACTGATGCTCGAAT |
| 1550526 | NM97192_snp14-F | TGCAAATCTTCAACCGCCAC |
|  | NM97192_snp14-R | ACGGTTTGGCTGCTTACCTT |
|  | NM97192_snp14S-F | CCATGCGTGCCTTTGTCTTT |
|  | NM97192_snp14S-R | TCTGTTGTACAATGCGCCCT |
| 1669031 | NM97192_snp16-F | CACGCCTCCAAAGACCTCAA |
|  | NM97192_snp16-R | GCAATCGGCAGGTGTTCTTC |
|  | NM97192_snp16S-F | GCAAGCGATTGATTGGCACA |
|  | NM97192_snp16S-R | CGACCAGCCACATCAAAAGC |
| 1997634 | NM97192_snp17-F | CGCTCTCACCGATAACCACA |
|  | NM97192_snp17-R | CTGTATGGTGCGCCGTCTTA |
|  | NM97192_snp17S-F | TTGGAAAGCAATCCTGCCGA |
|  | NM97192_snp17S-R | CGGATATGCCATGTTGACGC |
| 2041808 | anmk_F1 | ATCATGTCGGGAACCAGTATG |
|  | anmk_R1 | TTCACCGTTTTTGTCGTAAGG |
|  | anmk_seqR1 | GGAACACGGTTACAGCATACAG |

*Presence of “s” or “seq” indicates sequencing primers
